# A Comparison of the Risk of Viral Load Blips in Human Immunodeficiency Virus Patients on Two-Drug Versus Three-Drug Antiretroviral Regimens

**DOI:** 10.3390/idr17060141

**Published:** 2025-11-12

**Authors:** Kimihiro Yamaguchi, Masashi Ishihara, Yoshikazu Ikoma, Hitomi Sugiyama, Daichi Watanabe, Kei Fujita, Shin Lee, Tetsuji Morishita, Nobuhiro Kanemura, Masahito Shimizu, Hisashi Tsurumi

**Affiliations:** 1 Department of Hematology and Infectious Disease, Gifu University Hospital, Gifu 501-1194, Japan; 2 Department of Pharmacy, Gifu University Hospital, Gifu 501-1194, Japan; 3 AIDS Clinical Center, Gifu University Hospital, Gifu 501-1194, Japan; 4 Center for Nutrition Support and Infection Control, Gifu University Hospital, Gifu 501-1194, Japan; 5 Nursing Department, Gifu University Hospital, Gifu 501-1194, Japan; 6 Department of Hematology and Oncology, Matsunami General Hospital, Gifu 501-6062, Japan; 7 Department of Internal Medicine, Matsunami General Hospital, Gifu 501-6062, Japan

**Keywords:** antiretroviral therapy, HIV infection, viral blips, two-drug regimen, three-drug regimen, virologic suppression, generalized estimating equation, real-world cohort

## Abstract

**Background/Objectives**: The objective of this retrospective, multicenter cohort study was to compare the incidence of viral load blips between two-drug and three-drug antiretroviral therapy regimens in human immunodeficiency virus (HIV) patients. **Methods**: A total of 121 patients were included, with 44 receiving two-drug regimens (e.g., dolutegravir/lamivudine) and 77 receiving three-drug regimens (e.g., bictegravir/tenofovir alafenamide/emtricitabine) at the time of analysis. The primary outcome was the occurrence of viral blips, defined as transient HIV-RNA elevations ≥ 50 copies/mL; a sensitivity analysis used ≥20 copies/mL. **Results**: Generalized estimating equation models adjusted for clinical covariates showed no significant difference in the odds of blip occurrence comparing three-drug with two-drug regimens, both for blips ≥ 50 (odds ratio [OR]: 2.64; 95% confidence interval [CI]: 0.91–7.70; *p* = 0.075) and ≥20 (OR: 1.76; 95% CI: 0.76–4.08; *p* = 0.190). In the two- and three-drug groups, the predicted probabilities of blips were 1.4% and 3.7% (*p* = 0.075) for blips ≥ 50, and 6.9% and 11.5% (*p* = 0.190) for ≥20, respectively. No virologic failure was observed. **Conclusions**: These findings suggest that two-drug regimens provide virologic control comparable to three-drug regimens and may be a viable clinical option due to fewer drug interactions, lower toxicity, and reduced cost.

## 1. Introduction

The introduction of antiretroviral therapy (ART) has dramatically improved the prognosis and quality of life in human immunodeficiency virus (HIV)-1 infection [1,2]. Initially, triple-drug regimens dominated treatment, but adherence was often challenged by pill burden, tolerability issues, and resistance concerns [3]. Since the early 2010s, fixed-dose combinations such as elvitegravir/cobicistat/tenofovir disoproxil fumarate/emtricitabine (EVG/c/TDF/FTC) [4] and abacavir/lamivudine/dolutegravir (ABC/3TC/DTG) [5], followed by bictegravir/tenofovir alafenamide/emtricitabine (BIC/TAF/FTC) in 2019 [6], and long-acting injectable cabotegravir plus rilpivirine (CAB/RPV) in 2021 [7], have significantly streamlined treatment and improved patient adherence and outcomes.

Despite effective suppression of HIV-1 RNA below detection limits with ART, transient increases in viral load, termed “viral blips”, still occur in some patients. The clinical significance of blips remains controversial. Prospective studies have shown minimal clinical impact [8], whereas cohort analyses indicate associations with increased virologic failure risk [9,10], activation of latent HIV reservoirs [11], and prolonged half-life of latent reservoir cells [12]. Therefore, blips are generally regarded as events that should be avoided.

Recently, two-drug regimens, including dolutegravir/rilpivirine (DTG/RPV) and dolutegravir/lamivudine (DTG/3TC), have emerged and demonstrated comparable efficacy in maintaining viral suppression to traditional three-drug regimens in randomized, clinical trials [13,14,15,16]. Indeed, current international guidelines now recognize these two-drug regimens as suitable alternatives for initial and maintenance ART [17,18,19]. In addition, two-drug regimens offer potential advantages over three-drug regimens, including reduced risk of drug–drug interactions, lower medication costs, and fewer adverse events. However, detailed comparative data specifically evaluating the frequency and clinical features of viral blips between two-drug and three-drug regimens remain limited.

Therefore, this study aimed to compare the incidence and associated clinical characteristics of viral blips between patients receiving two-drug and three-drug ART regimens in a long-term, multicenter cohort.

## 2. Materials & Methods

### 2.1. Study Design and Patient Eligibility

This retrospective, multicenter, cohort study included patients who received ART at two institutions (Gifu University Hospital and Matsunami General Hospital) between 1 June 2004 and 31 May 2024. Patients were observed until 31 May 2025.

Patients were included if they were aged 18 years or older and had started ART between 1 June 2004 and 31 May 2024. Patients were excluded if they never achieved HIV-RNA levels below the detection limit (20 copies/mL) after initiating ART, had fewer than 10 HIV-RNA measurements or observation periods shorter than 6 months, exhibited significant medication nonadherence (based on the treating physician’s judgment), opted out of study participation, or were deemed unsuitable by the primary investigator. In addition, patients with significant comorbid conditions, coinfections, autoimmune diseases, or a history of injecting drug use that could affect ART continuation or viral blip risk were excluded at the discretion of the treating physician.

Patient data were anonymized before analysis. This study was conducted in accordance with the principles of the Declaration of Helsinki. All protocols were approved by the ethics committee of Gifu University Graduate School of Medicine (approval no. 2025-106, approved on 7 July 2025) under the centralized review system, and by the institutional review boards of each participating institution. The requirement for written, informed consent was waived in this study because the study exclusively used anonymized, retrospective data. All patients had an opt-out opportunity.

### 2.2. Data Collection

Clinical data were extracted from electronic medical records. Baseline characteristics included age, sex, acquired immune deficiency syndrome (AIDS) diagnosis according to U.S. Centers for Disease Control and Prevention (CDC) criteria (when applicable), baseline CD4 cell count (cells/µL), baseline HIV-RNA levels (copies/mL), duration of ART (years), treatment history, and history of drug resistance.

ART regimens were categorized into two groups according to the actual regimen prescribed at the time of analysis. Two-drug regimens included DTG/3TC, CAB/RPV, DTG/RPV, and DTG/doravirine (DTG/DOR). Three-drug regimens included BIC/TAF/FTC, ABC/3TC/DTG, TAF/FTC + raltegravir (RAL), TAF/FTC + darunavir (DRV)/c, DRV/c + DTG, ABC/3TC + RAL, TAF/FTC/DRV/c + RAL, TAF/FTC/DRV/c + DTG, TAF/FTC + DTG, and ABC/3TC + DTG.

Data collected regarding viral blips included the dates and values of HIV-RNA measurements, results of drug-resistance testing, and instances of treatment failure, defined as regimen changes or persistent detectable viral loads above the limit.

### 2.3. Definitions and Outcomes

The primary outcome was the occurrence of HIV viral load “blips,” defined as transient episodes of HIV-RNA ≥ 50 copies/mL following prior suppression below the detection limit. For sensitivity analysis, a lower threshold of ≥20 copies/mL was applied, reflecting the use of highly sensitive assays commonly adopted in clinical practice in Japan.

AIDS was defined according to the criteria of the CDC as the occurrence of any AIDS-defining illness or a CD4 cell count less than 200 cells/µL, as per the CDC revised classification and AIDS-defining conditions list [20].

### 2.4. Statistical Analysis

Baseline patient characteristics were summarized using medians with ranges for continuous variables and numbers with percentages for categorical variables. Comparisons between groups were performed using the Wilcoxon rank-sum test or Fisher’s exact test, as appropriate. Baseline characteristics were stratified into two groups (two-drug and three-drug regimens) according to the regimen patients were receiving at the end of the observation period, even if the regimen had been modified during follow-up.

The frequencies and counts of viral blips were tabulated for each ART regimen group. Generalized estimating equation (GEE) models with logit link were used to assess the association between potential risk factors and blip occurrence, accounting for repeated measurements per patient. Specifically, GEE was used to analyze the probability of experiencing a blip at each measurement point according to the ART regimen (two-drug or three-drug), adjusting for the following factors: age at the time of measurement, AIDS diagnosis at baseline, HIV-RNA level at ART initiation, CD4 cell count at ART initiation, and duration since ART initiation (years). Interactions between ART regimen types (two-drug vs. three-drug regimens) were tested within the GEE models. For interaction analyses, cut-off points were defined based on clinical guidelines and prior studies: age was dichotomized at 50 years (the threshold for “older adults with HIV”) [17,21], baseline HIV-RNA at 500,000 copies/mL (used in the GEMINI 1 & 2 trials) [13], CD4 count at 200 cells/µL (consistent with prophylaxis and AIDS-defining thresholds) [22], and duration since ART initiation at 2 years (as per long-term virologic outcome analyses) [23].

Univariable logistic regression models with restricted cubic splines (RCS) (4 knots at the 5th, 35th, 65th, and 95th percentiles) were applied to evaluate the nonlinear relationships of baseline HIV-RNA levels and CD4 cell counts with the incidence of viral blips.

Statistical analyses were conducted using R software (version 4.3.1, R Foundation for Statistical Computing, Vienna, Austria). A two-sided *p*-value < 0.05 was considered statistically significant.

## 3. Results

### 3.1. Patients’ Characteristics

A total of 121 patients were included in this study, with 44 patients in the two-drug regimen group and 77 patients in the three-drug regimen group. The patients’ baseline characteristics are summarized in Table 1. The majority of patients in both groups were male, with 88.6% in the two-drug group and 98.7% in the three-drug group, showing a significant difference (*p* = 0.024).

ART duration was significantly shorter in the two-drug group than in the three-drug group (median 5.0 vs. 8.2 years; *p* < 0.001). Age was slightly higher in the two-drug group than in the three-drug group (median 39.5 vs. 37 years; *p* = 0.042). Other baseline characteristics, including baseline HIV-RNA levels, CD4 cell counts, and the proportion of patients with an AIDS diagnosis, did not differ significantly between the groups. AIDS had been diagnosed in 17 of 44 patients (38.6%) in the two-drug group and 36 of 77 (46.8%) in the three-drug group (*p* = 0.448). The distributions of age (39.5 vs. 37 years; *p* = 0.042), baseline HIV-RNA (75,000 vs. 88,000 copies/mL; *p* = 0.105), and CD4 count (196.5 vs. 152 cells/μL; *p* = 0.731) were also comparable. Patients with higher baseline HIV-RNA levels were mainly found in the three-drug group, while CD4 count distributions appeared broadly similar between the groups (Supplementary Figure S1).

The antiretroviral regimens prescribed at the time of analysis are summarized in Table 2. In the two-drug regimen group, DTG/3TC was the most common regimen (n = 35). The three-drug regimen group showed greater diversity, with the most common regimen being BIC/TAF/FTC (n = 70).

### 3.2. Viral Blip Occurrence

The frequencies of viral blips stratified by ART regimen are summarized in Table 3. In the two-drug regimen group, viral blips ≥ 50 copies/mL occurred in 1.3% of all HIV-RNA measurements, and blips ≥ 20 copies/mL occurred in 5.3%. In contrast, in the three-drug regimen group, the corresponding frequencies were higher: 3.9% and 13.2%, respectively. In addition, we examined the number of patients who experienced viral blip events, counted based on the ART regimen that patients were receiving at the time of analysis, regardless of previous regimen (Supplementary Table S1). As shown in this analysis, no patients experienced ≥10 blips ≥ 50 copies/mL, whereas ≥10 blips ≥ 20 copies/mL occurred in 4 patients from the three-drug group and in none from the two-drug group.

**Figure 1:**
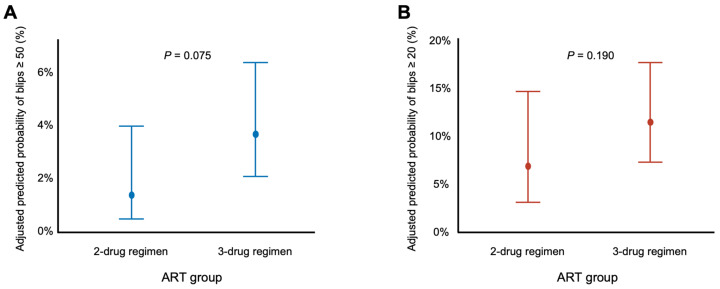
Predicted probability of viral blips by ART group. (**A**) Predicted probabilities of blips ≥ 50 copies/mL for patients on two-drug versus three-drug ART groups. (**B**) Predicted probabilities of blips ≥ 20 copies/mL for the same groups. Estimates were derived from GEE models adjusted for age, AIDS diagnosis, baseline HIV-RNA and CD4 count at ART initiation, and ART duration. Error bars represent 95% confidence intervals. ART, antiretroviral therapy; GEE, generalized estimating equation.

Generalized estimating equation (GEE) analysis was performed to evaluate factors associated with viral blip occurrences. For blips defined as HIV-RNA ≥ 50 copies/mL, patients receiving three-drug regimens had an odds ratio (OR) of 2.64 (95% confidence interval [CI]: 0.91–7.70; *p* = 0.075) compared with those receiving two-drug regimens, but this difference did not reach statistical significance (Table 4). The predicted probability of blips ≥ 50 copies/mL was 1.4% (95% CI: 0.5–4%) in the two-drug regimen group and 3.7% (95% CI: 2.1–6.4%) in the three-drug regimen group, but the difference was not significant (*p* = 0.075) (Figure 1A).

For blips defined as HIV-RNA ≥ 20 copies/mL, patients receiving three-drug regimens had an OR of 1.76 (95% CI: 0.76–4.08; *p* = 0.190) compared with those receiving two-drug regimens, but this difference did not reach statistical significance (Table 4). The predicted probability of blips ≥ 20 copies/mL was 6.9% (95% CI: 3.1–14.7%) in the two-drug regimen group and 11.5% (95% CI: 7.3–17.7%) in the three-drug regimen group, but the difference between groups was not significant (*p* = 0.190) (Figure 1B).

Other covariates included in the GEE model (age at measurement, AIDS diagnosis at baseline, HIV-RNA level at ART initiation, CD4 cell count at ART initiation, and duration since ART initiation) were not significantly associated with the occurrence of viral blips, regardless of the threshold used (≥50 or ≥20 copies/mL). Detailed results for all variables included in the GEE models are summarized in Table 4.

**Figure 2:**
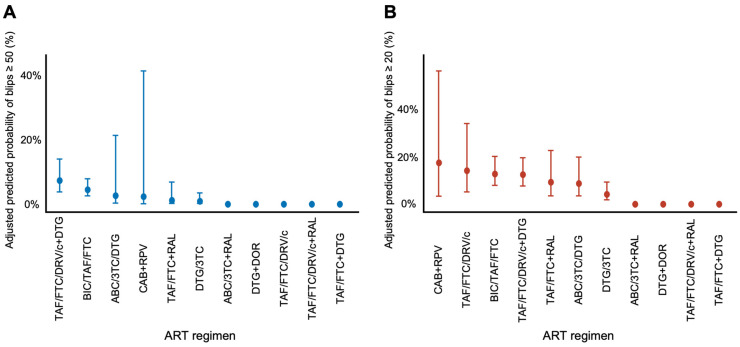
Predicted probabilities of viral blips by individual ART regimen (**A**) Predicted probabilities of blips ≥ 50 copies/mL for patients on individual ART regimens. (**B**) Predicted probabilities of blips ≥ 20 copies/mL for the same regimens. Estimates were derived from GEE models adjusted for age, AIDS diagnosis, baseline HIV-RNA and CD4 count at ART initiation, and ART duration. Error bars represent 95% confidence intervals. Some ART regimens may not be shown in the figure due to complete case exclusion in the GEE model (i.e., missing covariates). ART, antiretroviral therapy; ABC, abacavir; BIC, bictegravir; CAB, cabotegravir; c, cobicistat; DTG, dolutegravir; DOR, doravirine; DRV, darunavir; FTC, emtricitabine; RAL, raltegravir; RPV, rilpivirine; TAF, tenofovir alafenamide; 3TC, lamivudine.

To further explore differences in blip risk across individual ART regimens, predicted probabilities of blips (≥50 and ≥20 copies/mL) were estimated for each regimen using adjusted GEE models (Figure 2). The results demonstrated variability among regimens, but wide confidence intervals limited interpretability for less frequently used combinations. Of the most commonly prescribed regimens, DTG/3TC and BIC/TAF/FTC showed low predicted probabilities for both blip thresholds. The estimated probabilities for DTG/3TC were 0.9% (95% CI: 0.2–3.5%) for blips ≥ 50 copies/mL and 6.4% (95% CI: 3.2–12.2%) for blips ≥ 20 copies/mL, whereas those for BIC/TAF/FTC were 4.5% (95% CI: 2.6–7.9%) and 12.8% (95% CI: 7.9–20.1%), respectively. These findings suggest that regimens commonly used in clinical practice were generally associated with a low blip risk.

To evaluate potential interactions between ART regimen type (two-drug vs. three-drug) and patient-level factors associated with blip occurrence, subgroup analyses were performed using GEE models (Table 5). For blips ≥ 50 copies/mL, three-drug regimens were associated with higher odds of blips in patients aged ≥ 50 years (OR: 3.47; 95% CI: 1.29–9.29; *p* = 0.013) and those with CD4 < 200 cells/μL at ART initiation (OR: 4.01; 95% CI: 1.34–12.00; *p* = 0.013). Similarly, for blips ≥ 20 copies/mL, three-drug regimens showed increased odds in older patients (OR: 3.27; 95% CI: 1.53–6.99; *p* = 0.002) and in those with low CD4 counts (OR: 2.53; 95% CI: 1.30–4.91; *p* = 0.006). However, none of the interaction terms between regimen type and these subgroups was significant, indicating that the effect of ART regimen on blip risk did not significantly differ by age, CD4 count, or other baseline factors.

**Figure 3:**
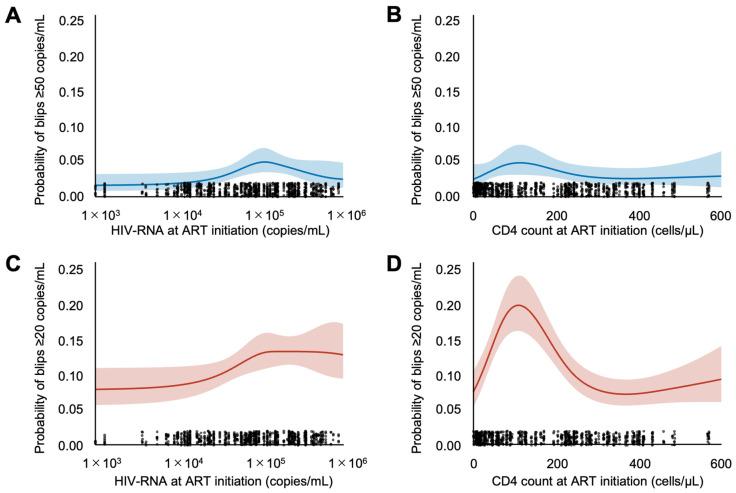
Predicted probability of viral blips according to baseline HIV-RNA or CD4 cell count at ART initiation based on RCS models. (**A**) Predicted probability of blips ≥ 50 copies/mL by HIV-RNA at ART initiation. (**B)** Predicted probability of blips ≥ 50 copies/mL by CD4 cell count at ART initiation. (**C**) Predicted probability of blips ≥ 20 copies/mL by HIV-RNA at ART initiation. (**D**) Predicted probability of blips ≥ 20 copies/mL by CD4 cell count at ART initiation. Restricted cubic spline (RCS) models with 4 knots (at the 5th, 35th, 65th, and 95th percentiles) were used to evaluate the nonlinear associations. Shaded areas represent 95% confidence intervals. Observed data points are plotted along the x-axis as rugs. RCS, restricted cubic spline; ART, antiretroviral therapy.

Using RCS models, we evaluated how baseline HIV-RNA and CD4 counts at ART initiation were nonlinearly associated with the probability of subsequent viral blips, as shown in Figure 3. The predicted probability of blips remained relatively stable at lower baseline HIV-RNA levels (<10^4^ copies/mL) and increased beyond this threshold. In addition, lower CD4 counts at ART initiation (<200 cells/µL) were associated with an increased probability of viral blips, with the risk progressively decreasing as CD4 counts increased above this threshold. To further explore whether these associations differed by regimen type, we repeated the analyses separately for patients on two- and three-drug regimens (Supplementary Figure S2). The overall trends were broadly similar across groups; however, interaction tests indicated some statistically significant differences, with several curves showing higher blip probabilities in the three-drug group. These subgroup analyses were exploratory and unadjusted, and thus the findings should be interpreted with caution.

No cases of virological treatment failure were observed during the study period.

## 4. Discussion

The present study demonstrated that the risk of viral load blips did not differ significantly between patients on two-drug regimens and those on three-drug regimens after adjusting for key confounders. In the GEE models, two-drug therapy was not associated with a higher likelihood of blips overall. Though subgroup analyses showed numerically higher blip risk with three-drug regimens in certain subgroups, such as older patients and those with low CD4 counts at ART initiation, interactions were not significant, suggesting that the effect of regimen type did not differ significantly across patient subgroups. These findings should be interpreted with caution given the limited sample size and wide confidence intervals. Furthermore, the restricted cubic spline analysis showed that baseline HIV-RNA levels and CD4 counts were important predictors of blip occurrence: patients with higher initial viral loads and lower CD4 counts at ART initiation had an increased probability of experiencing viral blips.

The present results align with growing evidence that modern two-drug regimens are as effective as traditional three-drug regimens in maintaining viral suppression. Randomized trials (GEMINI-1 and -2) demonstrated non-inferior efficacy of DTG/3TC dual therapy compared with standard triple therapy and durable suppression through three years. Notably, post hoc analyses of these trials reported no increase in intermittent viremia or “blips” with two-drug regimens. In the GEMINI trials, the incidence of viral blips was similar between the DTG/3TC arm and the three-drug arm, underscoring the durable efficacy of dual therapy [16]. Likewise, in the TANGO switch study, rates of having ≥1 viral load ≥50 copies were comparable between those who switched to DTG/3TC and those who continued triple therapy (4.9% vs. 7.0% over 144 weeks) [15]. These findings suggest that two-drug regimens, when composed of potent agents with high genetic barriers (e.g., integrase inhibitors with lamivudine or rilpivirine), can suppress HIV replication as robustly as three-drug regimens even in terms of low-level viremia. In the present cohort, no patient experienced virologic failure during follow-up, reinforcing the notion that isolated blips during effective therapy do not necessarily foreshadow treatment failure (consistent with prior prospective observations of minimal clinical impact from occasional blips) [8]. Overall, the lack of excess blip risk on two-drug ART in the present real-world setting provides reassurance that regimen simplification does not compromise short-term virologic control, a conclusion that is supported by both clinical trial data and other cohorts [24].

There was no difference in blip risk between two-drug and three-drug ART consistently across various patient subgroups. There was no evidence that factors such as age, sex, baseline disease status, or time on ART modified the effect of regimen type on blips, as indicated by non-significant interaction *p*-values in the GEE models. In other words, even patients traditionally considered at higher risk for viremia events, such as those older in age, with a history of AIDS, or with high baseline viral load or low CD4 counts, did not show a disproportionate increase in blips on two-drug therapy compared with three-drug therapy. This broad consistency suggests that the virologic performance of two-drug regimens is robust across different clinical scenarios. Similarly, subgroup analyses in the GEMINI study indicated no significant loss of virologic suppression with two-drug therapy, even in participants with baseline HIV-RNA > 100,000 copies or CD4 counts ≤ 200/µL [16]. Although those with more advanced HIV disease had numerically more blips in some analyses, this did not translate into higher failure rates [16]. The present real-world data, together with trial results, support that two-drug regimens maintain their efficacy across diverse patient populations, without any subgroup requiring a three-drug regimen to prevent blips.

The analysis of baseline virologic and immunologic factors provides additional insight into the nature of viral blips. There was a clear association between high pre-treatment viral load and the likelihood of blips. Patients whose baseline HIV-RNA exceeded approximately 10^4^ copies/mL were more prone to subsequent blip events, whereas those who started ART with lower viral loads had a relatively stable suppression profile. This finding is in line with previous reports from large cohorts showing that individuals with higher baseline viremia are at greater risk of experiencing viral blips [25]. One explanation is that a higher initial viral burden reflects a larger reservoir of latently infected cells or residual viral replication, making intermittent viral “leaks” more likely during therapy. A low CD4 T-cell count at ART initiation (<200 cells/µL) was also associated with a higher blip frequency, with the risk diminishing as CD4 counts improved beyond this threshold. Advanced immune suppression may impair the body’s ability to contain transient viral recrudescence early in treatment. Encouragingly, as immune reconstitution occurs with effective ART (rising CD4 counts), the propensity for blips appears to decline [16]. Although blips have not been conclusively linked to clinical failure, they may reflect stochastic activation of the latent reservoir or low-level viral replication, which could have implications for long-term cure strategies [11,12,26]. These findings highlight the importance of early treatment initiation; by starting ART before extensive viral replication and CD4 depletion occur, the risk of subsequent blips can be minimized [23]. They also emphasize that viral blips are a multifactorial phenomenon influenced by the pre-treatment virologic set point and immune status, in addition to adherence and drug potency.

This study has several limitations. First, as a retrospective, cohort study, the analysis was subject to the biases inherent in non-randomized data. Unmeasured confounders (such as varying adherence levels or provider practices) may have influenced blip occurrences, and causality cannot be definitively established. Second, the sample size, particularly the two-drug regimen group (44 patients), was relatively modest. This confers limited statistical power to detect small differences or rare outcomes; a true difference in blip rates between regimens might not have been apparent given the sample size. Therefore, the relatively small sample size, despite being drawn from two institutions, limits the generalizability of our findings and increases the possibility of type II error. In addition, although all patients were required to have at least 10 HIV-RNA measurements be included, longer observation periods naturally increase the likelihood of detecting viral blip events. In our cohort, the ART duration was shorter in the two-drug group compared with the three-drug group; therefore, if patients in the two-drug group had been observed for the same duration, the number of blips detected might have been different. Third, medication adherence was not directly measured. Blips can be precipitated by sporadic non-adherence leading to transient viremia [25], so the lack of adherence data means that blips caused by biological factors could not be differentiated from those potentially due to missed doses [27]. Fourth, the range of regimens represented was somewhat narrow. In the present cohort, DTG/3TC and BIC/FTC/TAF were the predominant regimens in the two-drug and three-drug groups, respectively. In fact, a large proportion of patients on two-drug therapy were receiving DTG/3TC, whereas the three-drug group was heavily weighted toward integrase-inhibitor-based regimens, including many on bictegravir-containing therapy. As a result, the present findings essentially reflect a comparison between DTG/3TC and BIC/TAF/FTC, with relatively few patients on other two-drug combinations (such as DTG/RPV) or older three-drug regimens. This may limit the generalizability of the conclusions to regimens not examined in the present study. Nonetheless, it is worth noting that DTG/3TC and BIC/TAF/FTC are among the most widely used regimens in contemporary HIV treatment [28,29]. These two regimens are recommended as preferred options in current treatment guidelines and have been widely adopted in real-world practice. Thus, although the present data are mainly limited to DTG/3TC and BIC/TAF/FTC, the results are highly relevant to today’s treatment landscape [17]. Last, this study did not evaluate ART selection in the context of comorbid conditions such as hepatitis B virus (HBV) infection. Importantly, current guidelines recommend that patients with HBV coinfection receive ART regimens that include tenofovir (TDF or TAF), rendering two-drug regimens without tenofovir inappropriate in such cases [17]. Furthermore, for individuals who are negative for hepatitis B surface antigen and lack hepatitis B surface antibodies, HBV vaccination is advised before initiating regimens that do not include tenofovir [17,22]. These clinical considerations should guide regimen selection based not only on the risk of viral blips, but also on broader comorbidity profiles.

In conclusion, the present multicenter cohort study found no significant difference in the incidence of viral load blips between two-drug and three-drug antiretroviral regimens. Two-drug ART maintained viral suppression as stably as three-drug therapy in the present real-world patient population, without an increase in transient viremia events. In addition to comparable viral suppression, two-drug regimens offer practical advantages such as reduced risk of drug–drug interactions, lower treatment costs, and fewer adverse events, making them an attractive option when blip risk is not elevated. Clinicians can be cautiously optimistic that switching suitable patients to a two-drug regimen or initiating treatment with integrase-based dual therapy will not compromise virologic stability in terms of viral blips. Further research in larger cohorts and diverse settings is warranted to confirm these findings, to assess their applicability to other two-drug strategies such as long-acting injectables or NRTI-sparing regimens, and to continue monitoring the long-term outcomes of two-drug regimen strategies.

## Figures and Tables

**Table 1:** Patients’ characteristics at ART initiation.

Factor	All Patients (n = 121)	Two-Drug Regimen (n = 44)	Three-Drug Regimen (n = 77)	*p*-Value *
Age, y [range]	37 [22–73]	39.5 [24–73]	37 [22–65]	0.042
Male—n (%)	115 (95.0)	39 (88.6)	76 (98.7)	0.024
Baseline HIV-RNA levels, copies/mL [range]	87,500 [320–7,100,000]	75,000 [320–750,000]	88,000 [1000–7,100,000]	0.105
Baseline CD4 cell count, cells/μL [range]	162 [0–871]	196.5 [1–566]	152 [0–871]	0.731
AIDS diagnosis—n (%)	53 (43.8)	17 (38.6)	36 (46.8)	0.448
Duration of ART, y [range]	7.7 [0.9–17.0]	5.0 [0.9–14.3]	8.2 [3.9–17.0]	<0.001

Patients were grouped based on the ART regimen prescribed at the time of analysis, regardless of previous regimen changes during the follow-up period. * *p*-values were calculated using the Wilcoxon rank-sum test for continuous variables and Fisher’s exact test for categorical variables. ART, antiretroviral therapy.

**Table 2:** Distribution of ART regimens at the time of analysis (N = 121).

ART Regimen	Patients, n (%)
Two-drug regimen	44 (36.4)
DTG/3TC	35 (28.9)
CAB/RPV	5 (4.1)
DTG/DOR	3 (2.5)
DTG/RPV	1 (0.8)
Three-drug regimen	77 (63.6)
BIC/TAF/FTC	70 (57.9)
TAF/FTC + RAL	3 (2.5)
ABC/3TC/DTG	1 (0.8)
TAF/FTC/DRV/c + RAL	1 (0.8)
TAF/FTC/DRV/c + DTG	1 (0.8)
ABC/3TC + RAL	1 (0.8)

The table presents the number of study participants using each specific ART regimen at the time of data analysis. ART, antiretroviral therapy; ABC, abacavir; BIC, bictegravir; CAB, cabotegravir; c, cobicistat; DTG, dolutegravir; DOR, doravirine; DRV, darunavir; FTC, emtricitabine; RAL, raltegravir; RPV, rilpivirine; TAF, tenofovir alafenamide; 3TC, lamivudine.

**Table 3:** Incidence of HIV viral blips by ART group and regimen.

ART Regimen	Total Tests	Blips ≥ 50, n (%)	Blips ≥ 20, n (%)
Two-drug regimen	546	7 (1.3)	29 (5.3)
DTG/3TC	455	5 (1.1)	18 (4.0)
CAB/RPV	62	2 (3.2)	11 (17.7)
DTG/DOR	16	0 (0.0)	0 (0.0)
DTG/RPV	13	0 (0.0)	0 (0.0)
Three-drug regimen	1620	63 (3.9)	214 (13.2)
BIC/TAF/FTC	1272	57 (4.5)	179 (14.1)
TAF/FTC + RAL	114	2 (1.8)	14 (12.3)
ABC/3TC/DTG	80	3 (3.8)	11 (13.8)
TAF/FTC/DRV/c	79	0 (0.0)	7 (8.9)
DRV/c + DTG	23	0 (0.0)	1 (4.3)
TAF/FTC/DRV/c + RAL	18	0 (0.0)	0 (0.0)
TAF/FTC/DRV/c + DTG	16	1 (6.2)	2 (12.5)
ABC/3TC + RAL	14	0 (0.0)	0 (0.0)
TAF/FTC + DTG	4	0 (0.0)	0 (0.0)

The table presents the total number of HIV-RNA measurements and the frequency and proportion of virological blips for each ART regimen, defined as ≥50 and ≥20 copies/mL. Blip frequency is expressed as the number of blips, and blip proportion is calculated as the percentage of blips out of the total HIV-RNA measurements for each regimen. ART, antiretroviral therapy; ABC, abacavir; BIC, bictegravir; CAB, cabotegravir; c, cobicistat; DTG, dolutegravir; DOR, doravirine; DRV, darunavir; FTC, emtricitabine; RAL, raltegravir; RPV, rilpivirine; TAF, tenofovir alafenamide; 3TC, lamivudine.

**Table 4:** Predictors of viral blips in GEE models.

Factor	Odds Ratio (95% CI)	*p*-Value
Blips ≥ 50 copies/mL		
Three-drug regimen	2.64 (0.91–7.70)	0.075
Age	1.02 (0.98–1.06)	0.278
AIDS diagnosis	0.78 (0.25–2.49)	0.679
HIV-RNA at ART initiation	1.00 (1.00–1.00)	0.455
CD4 cell count at ART initiation	1.00 (1.00–1.00)	0.476
Duration of ART (y)	0.92 (0.81–1.04)	0.162
Blips ≥ 20 copies/mL		
Three-drug regimen	1.76 (0.76–4.08)	0.190
Age	1.01 (0.99–1.04)	0.317
AIDS diagnosis	0.78 (0.30–2.02)	0.613
HIV-RNA at ART initiation	1.00 (1.00–1.00)	0.881
CD4 cell count at ART initiation	1.00 (1.00–1.00)	0.246
Duration of ART (y)	0.87 (0.79–0.95)	0.003

The table presents the results from GEE models examining factors associated with blips defined as ≥50 and ≥20 copies/mL separately. Reference category for the ART group is the two-drug regimen. Adjusted for age, AIDS at diagnosis, baseline HIV-RNA, baseline CD4 count, and treatment duration. ART, antiretroviral therapy; CI, confidence interval; GEE, generalized estimating equation.

**Table 5:** Interaction effects of patient subgroups on viral blips in GEE models.

Subgroup	N	Odds Ratio (95% CI)	*p*-Value	*P* for Interaction
Blips ≥ 50 copies/mL				
AIDS diagnosis				0.987
Yes	50	2.80 (0.70–11.24)	0.148	
No	64	2.86 (0.59–13.82)	0.191	
Age (y)				0.642
<50	79	2.17 (0.24–19.53)	0.489	
≥50	58	3.47 (1.29–9.29)	0.013	
HIV-RNA at ART initiation				NA
<500,000	100	2.44 (0.81–7.37)	0.113	
≥500,000	14	NA	NA	
CD4 cell count at ART initiation				0.592
<200	62	4.01 (1.34–12.00)	0.013	
≥200	52	1.92 (0.21–17.22)	0.559	
Duration of ART				NA
<2 y	16	NA	NA	
≥2 y	114	NA	NA	
Blips ≥ 20 copies/mL				
AIDS diagnosis				0.5
Yes	50	2.84 (0.91–8.83)	0.071	
No	64	1.58 (0.52–4.84)	0.423	
Age (y)				0.171
<50	79	1.20 (0.36–4.04)	0.763	
≥50	58	3.27 (1.53–6.99)	0.002	
HIV-RNA at ART initiation				0.526
<500,000	100	1.75 (0.75–4.04)	0.193	
≥500,000	14	5.36 (0.06–502)	0.468	
CD4 cell count at ART initiation				0.954
<200	62	2.53 (1.30–4.91)	0.006	
≥200	52	1.38 (0.24–7.93)	0.716	
Duration of ART				0.362
<2 y	16	1.11 (0.40–3.06)	0.846	
≥2	114	2.06 (0.82–5.20)	0.126	

Odds ratios indicate the relative odds of viral blips in patients receiving three-drug regimens compared with those on two-drug regimens. NA, not available; estimates could not be calculated because the number of events in the subgroup was extremely small and the model did not converge. ART, antiretroviral therapy; CI, confidence interval; GEE, generalized estimating equation.

## Data Availability

The anonymized patient data sets and statistical code analyzed in this study are available from the corresponding author upon reasonable request.

## Supplementary Materials

The following supporting information can be downloaded at https://www.mdpi.com/article/10.3390/idr17060141/s1, Figure S1: Baseline HIV-RNA and CD4 by ART group, Figure S2: Predicted probability of viral blips according to baseline HIV-RNA and CD4 cell count at ART initiation based on RCS models, stratified by regimen type (two-drug vs. three-drug); Table S1: Patients with viral blip events and HIV-RNA levels by ART group.

## References

[1] Antiretroviral Therapy Cohort Collaboration (2017). Survival of HIV-positive patients starting antiretroviral therapy between 1996 and 2013: A collaborative analysis of cohort studies. Lancet HIV.

[2] Dutra B.S., Lédo A.P., Lins-Kusterer L., Luz E., Prieto I.R., Brites C. (2019). Changes health-related quality of life in HIV-infected patients following initiation of antiretroviral therapy: A longitudinal study. Braz. J. Infect. Dis..

[3] Tseng A., Seet J., Phillips E.J. (2015). The evolution of three decades of antiretroviral therapy: Challenges, triumphs and the promise of the future: Three decades of antiretroviral therapy. Br. J. Clin. Pharmacol..

[4] Rockstroh J.K., DeJesus E., Henry K., Molina J.-M., Gathe J., Ramanathan S., Wei X., Plummer A., Abram M., Cheng A.K. (2013). A randomized, double-blind comparison of coformulated elvitegravir/cobicistat/emtricitabine/tenofovir DF vs ritonavir-boosted atazanavir plus coformulated emtricitabine and tenofovir DF for initial treatment of HIV-1 infection: Analysis of week 96 results: Analysis of week 96 results. J. Acquir. Immune Defic. Syndr..

[5] Walmsley S.L., Antela A.A., Clumeck N., Duiculescu D., Eberhard A.A., Gutiérrez F., Hocqueloux L.L., Maggiolo F.F., Sandkovsky U.U., Granier C.C. (2013). Dolutegravir plus abacavir-lamivudine for the treatment of HIV-1 infection. N. Engl. J. Med..

[6] Sax P.E., Arribas J.R., Orkin C., Lazzarin A., Pozniak A., DeJesus E., Maggiolo F., Stellbrink H.-J., Yazdanpanah Y., Acosta R. (2023). Bictegravir/emtricitabine/tenofovir alafenamide as initial treatment for HIV-1: Five-year follow-up from two randomized trials. EClinicalMedicine.

[7] Mills A., Richmond G.J., Newman C., Osiyemi O., Cade J., Brinson C., De Vente J., Margolis D.A., Sutton K.C., Wilches V. (2022). Long-acting cabotegravir and rilpivirine for HIV-1 suppression: Switch to 2-monthly dosing after 5 years of daily oral therapy. AIDS.

[8] Nettles R.E., Kieffer T.L., Kwon P., Monie D., Han Y., Parsons T., Cofrancesco J., Gallant J.E., Quinn T.C., Jackson B. (2005). Intermittent HIV-1 viremia (Blips) and drug resistance in patients receiving HAART. JAMA.

[9] Young J., Rickenbach M., Calmy A., Bernasconi E., Staehelin C., Schmid P., Cavassini M., Battegay M., Günthard H.F., Bucher H.C. (2015). Transient detectable viremia and the risk of viral rebound in patients from the Swiss HIV Cohort Study. BMC Infect. Dis..

[10] Elvstam O., Malmborn K., Elén S., Marrone G., García F., Zazzi M., Sönnerborg A., Böhm M., Seguin-Devaux C., Björkman P. (2023). Virologic failure following low-level viremia and viral blips during antiretroviral therapy: Results from a European multicenter cohort. Clin. Infect. Dis..

[11] Suzuki K., Levert A., Yeung J., Starr M., Cameron J., Williams R., Rismanto N., Stark T., Druery D., Prasad S. (2021). HIV-1 viral blips are associated with repeated and increasingly high levels of cell-associated HIV-1 RNA transcriptional activity. AIDS.

[12] Siliciano J.D., Kajdas J., Finzi D., Quinn T.C., Chadwick K., Margolick J.B., Kovacs C., Gange S., Siliciano R.F. (2003). Long-term follow-up studies confirm the stability of the latent reservoir for HIV-1 in resting CD4+ T cells. Nat. Med..

[13] Cahn P., Madero J.S., Arribas J.R., Antinori A., Ortiz R., Clarke A.E., Hung C.-C., Rockstroh J.K., Girard P.-M., Sievers J. (2019). Dolutegravir plus lamivudine versus dolutegravir plus tenofovir disoproxil fumarate and emtricitabine in antiretroviral-naive adults with HIV-1 infection (GEMINI-1 and GEMINI-2): Week 48 results from two multicentre, double-blind, randomised, non-inferiority, phase 3 trials. Lancet.

[14] Cahn P., Madero J.S., Arribas J.R., Antinori A., Ortiz R., Clarke A.E., Hung C.-C., Rockstroh J.K., Girard P.-M., Sievers J. (2022). Three-year durable efficacy of dolutegravir plus lamivudine in antiretroviral therapy—Naive adults with HIV-1 infection. AIDS.

[15] Wang R., Underwood M., Llibre J.M., Morell E.B., Brinson C., Moreno J.S., Scholten S., Moore R., Saggu P., Oyee J. (2024). Very-low-level viremia, inflammatory biomarkers, and associated baseline variables: Three-year results of the randomized TANGO study. Open Forum Infect. Dis..

[16] Underwood M., Urbaityte R., Wang R., Horton J., Oyee J., Wynne B., Fox D., Jones B., Man C., Sievers J. (2024). Dolutegravir + lamivudine vs. Dolutegravir + tenofovir disoproxil fumarate/emtricitabine: Very-low-level HIV-1 replication through 144 weeks in the GEMINI-1 and GEMINI-2 studies. Viruses.

[17] Panel on Antiretroviral Guidelines for Adults and Adolescents Guidelines for the Use of Antiretroviral Agents in Adults and Adolescents with HIV. https://clinicalinfo.hiv.gov/sites/default/files/guidelines/documents/adult-adolescent-arv/guidelines-adult-adolescent-arv.pdf.

[18] Sterling T.R., Njie G., Zenner D., Cohn D.L., Reves R., Ahmed A., Menzies D., Horsburgh C.R., Crane C.M., Burgos M. (2020). Guidelines for the treatment of latent tuberculosis infection: Recommendations from the National Tuberculosis Controllers Association and CDC, 2020. MMWR Recomm. Rep..

[19] Gandhi R.T., Bedimo R., Hoy J.F., Landovitz R.J., Smith D.M., Eaton E.F., Lehmann C., Springer S.A., Sax P.E., Thompson M.A. (2023). Antiretroviral drugs for treatment and prevention of HIV infection in adults: 2022 recommendations of the international antiviral society-USA panel: 2022 recommendations of the international antiviral society-USA panel. JAMA.

[20] Centers for Disease Control and Prevention (1992). 1993 revised classification system for HIV infection and expanded surveillance case definition for AIDS among adolescents and adults. MMWR Recomm. Rep..

[21] McMillan J.M., Krentz H., Gill M.J., Hogan D.B. (2018). Managing HIV infection in patients older than 50 years. CMAJ.

[22] Panel on Guidelines for the Prevention and Treatment of Opportunistic Infections in Adults and Adolescents With HIV Guidelines for the Prevention and Treatment of Opportunistic Infections in Adults and Adolescents. https://clinicalinfo.hiv.gov/sites/default/files/guidelines/archive/adult-adolescent-oi-2024-10-08.pdf.

[23] Crowell T.A., Hsieh H.-C., Wang X., Chu X., Gayle B., Berjohn C.M., Blaylock J.M., Yabes J.M., Larson D.T., Powers J.H. (2025). Antiretroviral therapy within two years of HIV acquisition is associated with fewer viral blips: A retrospective analysis of over 20 years of data from the U.S. military HIV Natural History Study. Clin. Infect. Dis..

[24] Fraysse J., Priest J., Turner M., Hill S., Jones B., Verdier G., Letang E. (2025). Real-world effectiveness and tolerability of dolutegravir and lamivudine 2-drug regimen in people living with HIV: Systematic literature review and meta-analysis. Infect. Dis. Ther..

[25] Sörstedt E., Nilsson S., Blaxhult A., Gisslén M., Flamholc L., Sönnerborg A., Yilmaz A. (2016). Viral blips during suppressive antiretroviral treatment are associated with high baseline HIV-1 RNA levels. BMC Infect. Dis..

[26] Oomen P.G.A., Dijkstra S., Hofstra L.M., Nijhuis M.M., Verbon A., Mudrikova T., Wensing A.M.J., Hoepelman A.I.M., Van Welzen B.J. (2023). Integrated analysis of viral blips, residual viremia, and associated factors in people with HIV: Results from a retrospective cohort study. J. Med. Virol..

[27] Manalel J.A., Kaufman J.E., Wu Y., Fusaris E., Correa A., Ernst J., Brennan-Ing M. (2024). Association of ART regimen and adherence to viral suppression: An observational study of a clinical population of people with HIV. AIDS Res. Ther..

[28] Hocqueloux L., Lefeuvre S., Bois J., Brucato S., Alix A., Valentin C., Peyro-Saint-Paul L., Got L., Fournel F., Dargere S. (2022). Bioavailability of dissolved and crushed single tablets of bictegravir, emtricitabine, tenofovir alafenamide in healthy adults: The SOLUBIC randomized crossover study. J. Antimicrob. Chemother..

[29] Taguchi N., Piao Y., Rubino A., Lee K., Chen M., Harada K., Tanikawa T., Naito T. (2024). Relationship between adherence to bictegravir/emtricitabine/tenofovir alafenamide fumarate and clinical outcomes in people with HIV in Japan: A claims database analysis. Sci. Rep..

