# Supplementary material for: A Comparison of the Risk of Viral Load Blips in Human Immunodeficiency Virus Patients on Two-Drug Versus Three-Drug Antiretroviral Regimens
Source: Infect Dis Rep. 2025 Nov 12;17(6):141. doi: 10.3390/idr17060141 (PMC12641778; doi:10.3390/idr17060141)
Supplement: Supplementary file 1 [file idr-17-00141-s001.zip › idr-3839179-supplementary.pdf]

Supplementary Data

Supplementary Figure S1

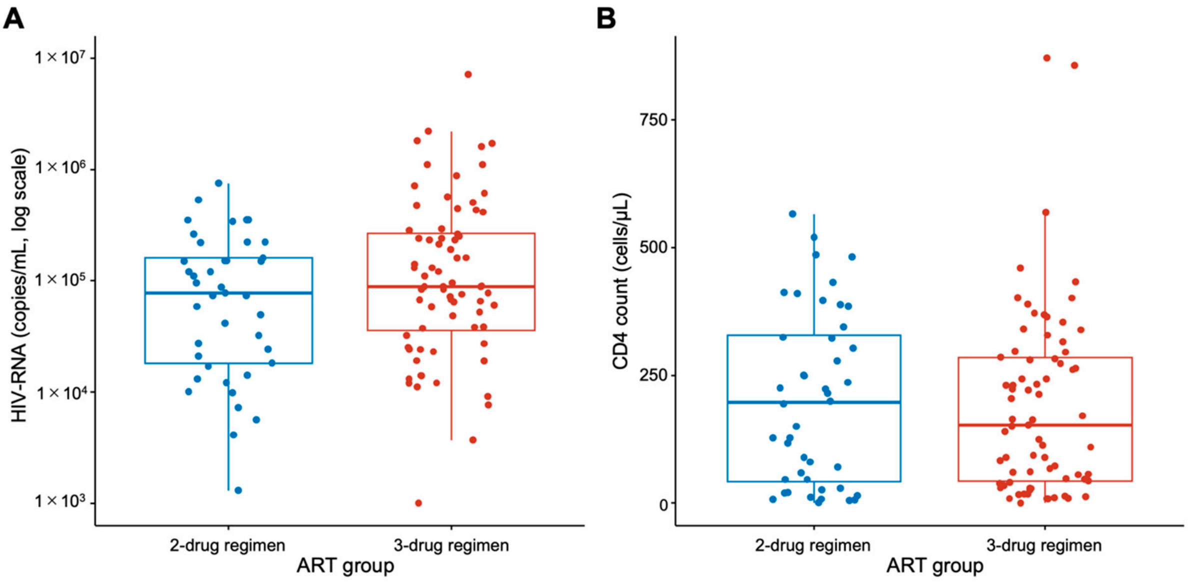

Supplementary Figure S1. Baseline HIV-RNA and CD4 by ART group.

A) Baseline HIV-RNA levels (copies/mL, $\log_{10}$ scale) for patients on two-drug versus three-drug ART.

B) Baseline CD4 cell counts (cells/μL) for the same groups; distributions appeared broadly similar.

In both panels, box plots show the median and interquartile range; whiskers denote 1.5×IQR; dots represent individual patients. ART, antiretroviral therapy.

Supplementary Figure S2

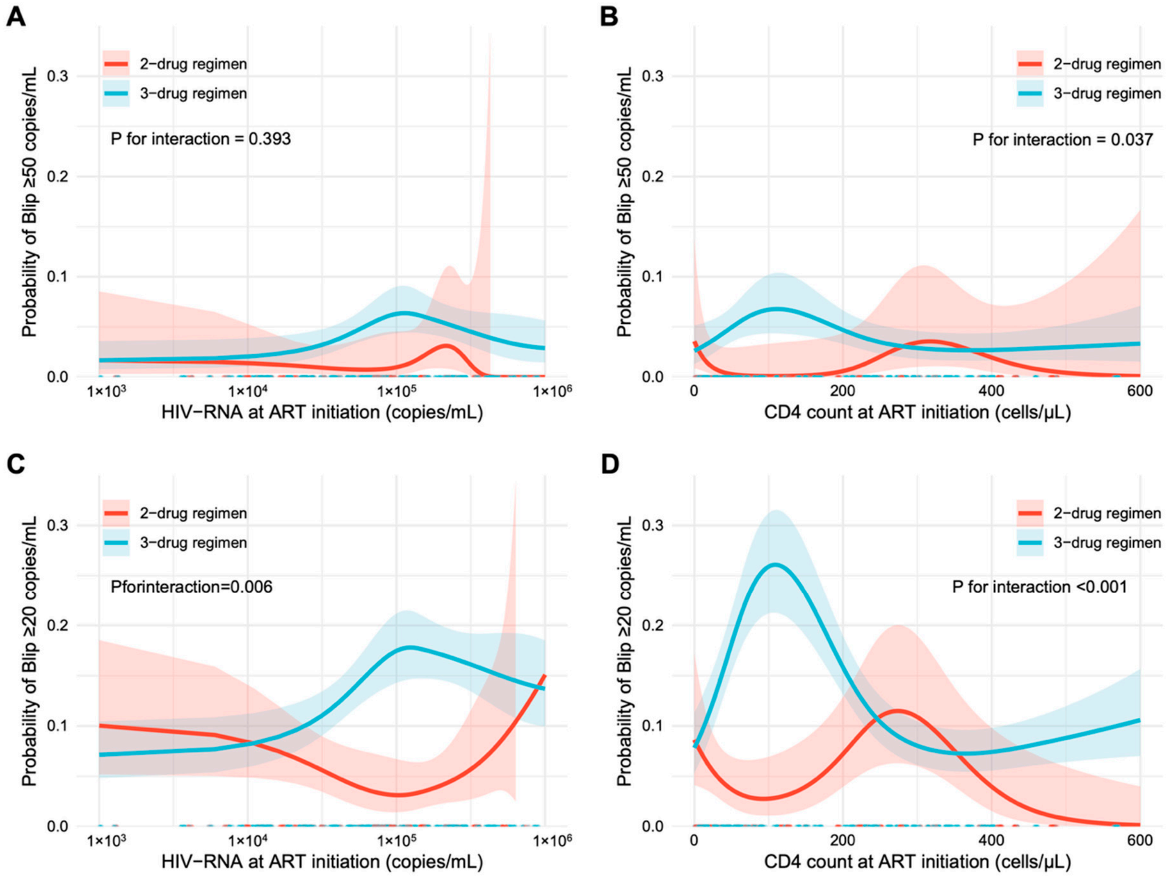

Supplementary Figure S2. Predicted probability of viral blips according to baseline HIV-RNA and CD4 cell count at ART initiation based on RCS models, stratified by regimen type (two-drug vs. three-drug).

A) Predicted probability of blips ≥ 50 copies/mL by baseline HIV-RNA.

B) Predicted probability of blips ≥ 50 copies/mL by baseline CD4 cell count.

C) Predicted probability of blips ≥ 20 copies/mL by baseline HIV-RNA.

D) Predicted probability of blips ≥ 20 copies/mL by baseline CD4 cell count.

Restricted cubic spline (RCS) models, with 4 knots, were fitted separately for the two- and three-drug groups, and interaction P-values were calculated to assess whether the associations differed by regimen type. Shaded areas represent 95% confidence intervals. Observed data points are plotted along the x-axis as rugs. RCS, restricted cubic spline; ART, antiretroviral therapy.

Supplementary Table S1. Patients with viral blip events and HIV-RNA levels by ART group

ART group	Blips ≥50, n (%)	HIV-RNA ≥50, copies/mL, median (range)	Blips ≥20, n (%)	HIV-RNA ≥20, copies/mL, median (range)
Two-drug regimen, n = 44	5 (11.4%)	87 (50–310)	11 (25.0%)	32 (20–310)
Three-drug regimen, n = 77	22 (28.6%)	76 (50–500)	39 (50.6%)	36 (20–500)

Number of patients with blip events is shown as n (%). HIV-RNA levels represent the median (range) of blip values among patients who experienced events. Blip events were counted based on the ART regimen that patients were receiving at the time of analysis, regardless of prior regimen changes during follow-up. The list of ART regimens included in each group is provided in Table 2.
ART, antiretroviral therapy.
